# Supplementary material for: Eicosapentaenoic Acid (EPA) Modulates Glucose Metabolism by Targeting AMP-Activated Protein Kinase (AMPK) Pathway
Source: Int J Mol Sci. 2019 Sep 25;20(19):4751. doi: 10.3390/ijms20194751 (PMC6801536; doi:10.3390/ijms20194751)
Supplement: Supplementary file 1 [file ijms-20-04751-s001.pdf]

Eicosapentaenoic Acid (EPA) Modulates Glucose Metabolism by Targeting AMP-activated Protein Kinase (AMPK) pathway

Nami Kim‡, Mi Sun Kang‡, Miso Nam‡, Shin Ae Kim§, Geum-Sook Hwang‡¶*, and Hyeon Soo Kim§*

‡Western Seoul Center, Korea Basic Science Institute, Seoul 120-140, Republic of Korea

§Department of Anatomy, Korea University College of Medicine, Seoul 136-701, Republic of Korea

¶Chemistry & Nanoscience, Ewha Womans University, Seoul 120-750, Republic of Korea

*Corresponding authors: Hyeon Soo Kim, MD, PhD, 126-1 Anam-dong 5-ga, Seongbuk-gu, Seoul, Korea. Tel: 82-2-2286-1151; Fax: 82-2-929-5696; E-mail: anatomykim@korea.ac.kr

Geum-Sook Hwang, PhD, 2-203 Shinchon-dong, Seodaemun-gu, Seoul, Korea. Tel: 82-2-6908-6200; Fax: 82-2-6908-6239; E-mail: gshwang@kbsi.re.kr

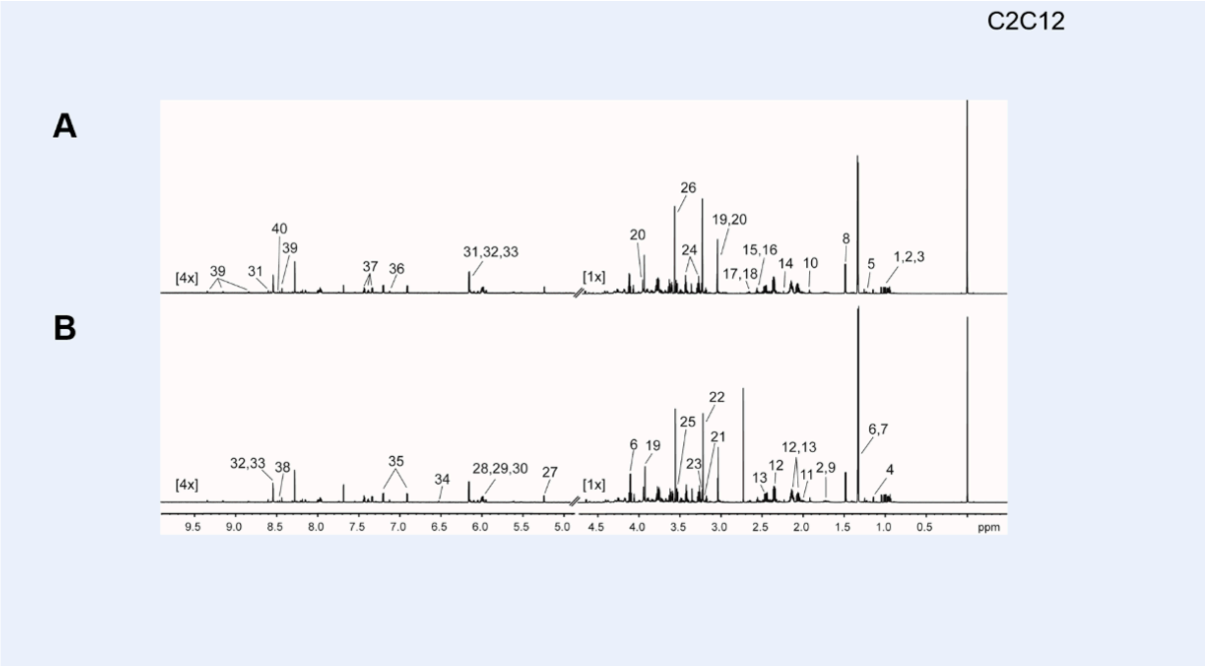

Supplementary Fig. 1

Supplementary Table 1

C2C12

Metabolite	Concentration mean ± SD (μM)		
	Control	EPA	*p*-value
valine	19.66 ± 0.63	22.86 ± 0.53	<0.001
leucine	20.9 ±0.51	23.32 ± 0.37	<0.001
isoleucine	21.6 ± 0.64	25 ± 0.48	<0.001
propylene glycol	24.72 ± 16.25	16.16 ± 5.48	0.297
ethanol	2.22 ± 0.2	2.2 ± 0.1	0.849
lactate	382.5 ±28.53	493.18 ± 12.95	<0.001
threonine	75.64 ± 1.55	79.76 ± 1.42	0.002
alanine	98.52 ± 3.5	98.3 ± 2.16	0.908
lysine	12.02 ± 0.58	13.38 ± 0.31	0.002
acetate	4.98 ± 0.81	5.76 ± 0.25	0.074
proline	16.64 ± 1.29	17.02 ± 0.26	0.535
glutamate	288.58 ± 9.72	286.96 ± 8.96	0.791
glutamine	172.6 ± 8.54	199.62 ± 3.04	<0.001
acetone	1.98 ± 0.04	2 ± 0.07	0.608
glutathione	19.5 ± 1.01	6.54 ± 0.89	<0.001
β-alanine	26.94 ± 0.95	26.78 ± 0.51	0.748
methionine	7.62 ± 0.19	9 ± 0.16	<0.001
malate	13.62 ± 0.18	12.88 ± 0.68	0.046
creatine	67.3 ± 2.24	70 ± 4.18	0.236
creatine phosphate	32.94 ± 2.92	30.08 ± 3.84	0.222

Metabolite	Concentration mean ± SD (μM)		
	Control	EPA	*p*-value
choline	0.74 ± 0.15	0.86 ± 0.23	0.359
O-phosphocholine	75.72 ± 1.79	71.64 ± 2.1	0.011
sn-glycero-3-phosphocholine	7.34 ± 0.36	8.62 ± 0.13	<0.001
taurine	100.08 ± 3.49	99.54 ± 2.03	0.772
myo-inositol	76.9 ± 2.25	78.06 ± 2.65	0.477
glycine	171.64 ± 6.23	180.38 ± 4.84	0.038
glucose	44.66 ± 4.12	47.32 ± 4.46	0.356
UDP-glucose	8.62 ± 0.24	8.4 ± 0.3	0.235
UDP-galactose	5.86 ± 0.15	5.4 ± 0.2	0.003
UDP-glucuronate	4.56 ± 0.24	4.08 ± 0.23	0.012
AMP	3.28 ± 0.63	5.58 ± 0.99	0.002
ADP	16.56 ± 1.16	19.2 ± 1.28	0.009
ATP	47.58 ± 2.61	41.76 ± 2.53	0.007
Fumarate	0.66 ± 0.55	0.6 ± 0.07	0.172
tyrosine	14.42 ± 0.44	16.64 ± 0.54	<0.001
histidine	4.4 ± 0.23	5.14 ± 0.19	0.001
phenylalanine	12.1 ± 0.4	13.94 ± 0.15	<0.001
formate	2.56 ± 0.46	2.32 ± 0.33	0.374
NAD+	6.14 ± 0.32	6.18 ± 0.13	0.803
NADH	1.52 ± 0.41	1.42 ± 0.33	0.681
